# Long-Term Survival Achieved by Combined Surgical Resection and Immunotherapy for Recurrent Adrenal Oligometastasis of Lung Adenocarcinoma: A Case Study

**DOI:** 10.7759/cureus.104958

**Published:** 2026-03-09

**Authors:** Sachie Hasegawa, Takumi Kiwamoto, Yuka Aida, Toshihiro Shiozawa, Nobuyuki Hizawa

**Affiliations:** 1 Department of Pulmonary Medicine, University of Tsukuba, Tsukuba, JPN

**Keywords:** adrenalectomy, adrenal gland metastasis, immune-checkpoint-inhibitor, immune checkpoint inhibitor (ici), long-term disease-free survival, long-term overall survival, lung adenocarcinoma (ladc), oligometastasis

## Abstract

A 73-year-old woman with epidermal growth factor receptor (EGFR) L858R-mutant lung adenocarcinoma (cT4N1M1a stage IV, programmed death-ligand 1 (PD-L1) high expression (60%)) received osimertinib as first-line therapy, but disease progression was observed after two months. She subsequently received second-line therapy with carboplatin, paclitaxel, and atezolizumab, followed by maintenance atezolizumab. During maintenance therapy, solitary metastasis developed in the left adrenal gland, while all other lesions remained well controlled. The condition was diagnosed as oligoprogression, and laparoscopic left adrenalectomy was performed. Histopathological examination confirmed adrenal metastasis from lung cancer. Atezolizumab was resumed 42 days after surgery, and the patient has remained recurrence-free for more than four years, achieving long-term survival.

With the widespread use of immune checkpoint inhibitors (ICIs), the prognosis of advanced non-small cell lung cancer has improved. However, the optimal treatment strategy for patients who develop localized progression (oligoprogression) during systemic therapy has not been established. This case demonstrates long-term survival achieved by combining surgical resection with resumption of ICI therapy for solitary adrenal oligoprogression during ICI maintenance therapy. The findings suggest that local treatment for limited progressive lesions during ICI therapy may be a useful therapeutic strategy.

## Introduction

With the widespread use of immune checkpoint inhibitors (ICIs) and the expansion of treatment options, including combination therapy with ICIs and platinum-based chemotherapy, clinical outcomes in patients with unresectable non-small cell lung cancer (NSCLC) have improved markedly [1,2]. In contrast, adrenal metastases from lung cancer have been suggested to exhibit a lower response rate to immunotherapy, similar to metastases to other organs [3].

As the concept of oligometastasis has become widely accepted, local treatment of metastatic lesions has been suggested to improve survival when other disease sites are controlled by systemic therapy [4,5]. However, there are few reports regarding the efficacy of local treatment for lesions resistant to ICI therapy during ongoing treatment [6]. Here, we report a case of solitary adrenal recurrence that developed during ICI maintenance therapy, in which long-term survival was achieved by surgical resection followed by resumption of ICI therapy, with a review of the relevant literature.

## Case presentation

A 73-year-old woman was admitted for treatment of recurrent left adrenal metastasis from lung adenocarcinoma, which had been treated for one year prior. She had no history of smoking and had been treated for hypertension.

**Figure 1:**
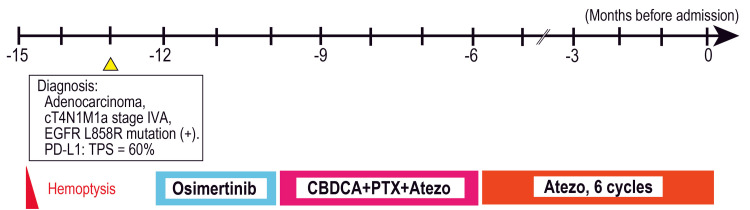
Clinical course Clinical course up to admission. EGFR: epidermal growth factor receptor; PD-L1: programmed death-ligand 1; TPS: tumor proportion score; CBDCA: carboplatin; PTX: paclitaxel; Atezo: atezolizumab. Image credit: Timeline image created by Dr. Takumi Kiwamoto with Adobe Illustrator 2026 (Adobe Systems, San Jose, CA).

At 72 years of age, she presented to our outpatient clinic after hemoptysis led to the detection of multiple tumors in the left lung, mediastinal lymphadenopathy, and pleural dissemination. Bronchoscopic examination and imaging studies resulted in a diagnosis of epidermal growth factor receptor (EGFR) L858R-mutant lung adenocarcinoma (cT4N1M1a stage IV, programmed death-ligand 1 (PD-L1) high expression (60%)). Osimertinib was initiated as first-line therapy one year prior to admission; however, computed tomography (CT) performed two months later showed a 20% increase in the sum of the diameters of the target lesions, with an absolute increase of 6 mm, consistent with progressive disease according to the Response Evaluation Criteria in Solid Tumors (RECIST) 1.1 criteria (Figure 1). A combination chemotherapy with carboplatin, paclitaxel, bevacizumab, and atezolizumab was initially considered. However, because hemoptysis persisted and cavitation was observed in the primary tumor, bevacizumab was withheld due to safety concerns, and second-line therapy was administered with carboplatin, paclitaxel, and atezolizumab.

Ten months prior to admission, a second-line therapy with carboplatin, paclitaxel, and atezolizumab was administered for four cycles, resulting in a trend toward tumor reduction of the primary lesion, pleural dissemination, and left hilar lymph node metastasis, although the response was classified as stable disease. After the completion of combination therapy, six cycles of maintenance atezolizumab were continued.

**Figure 2:**
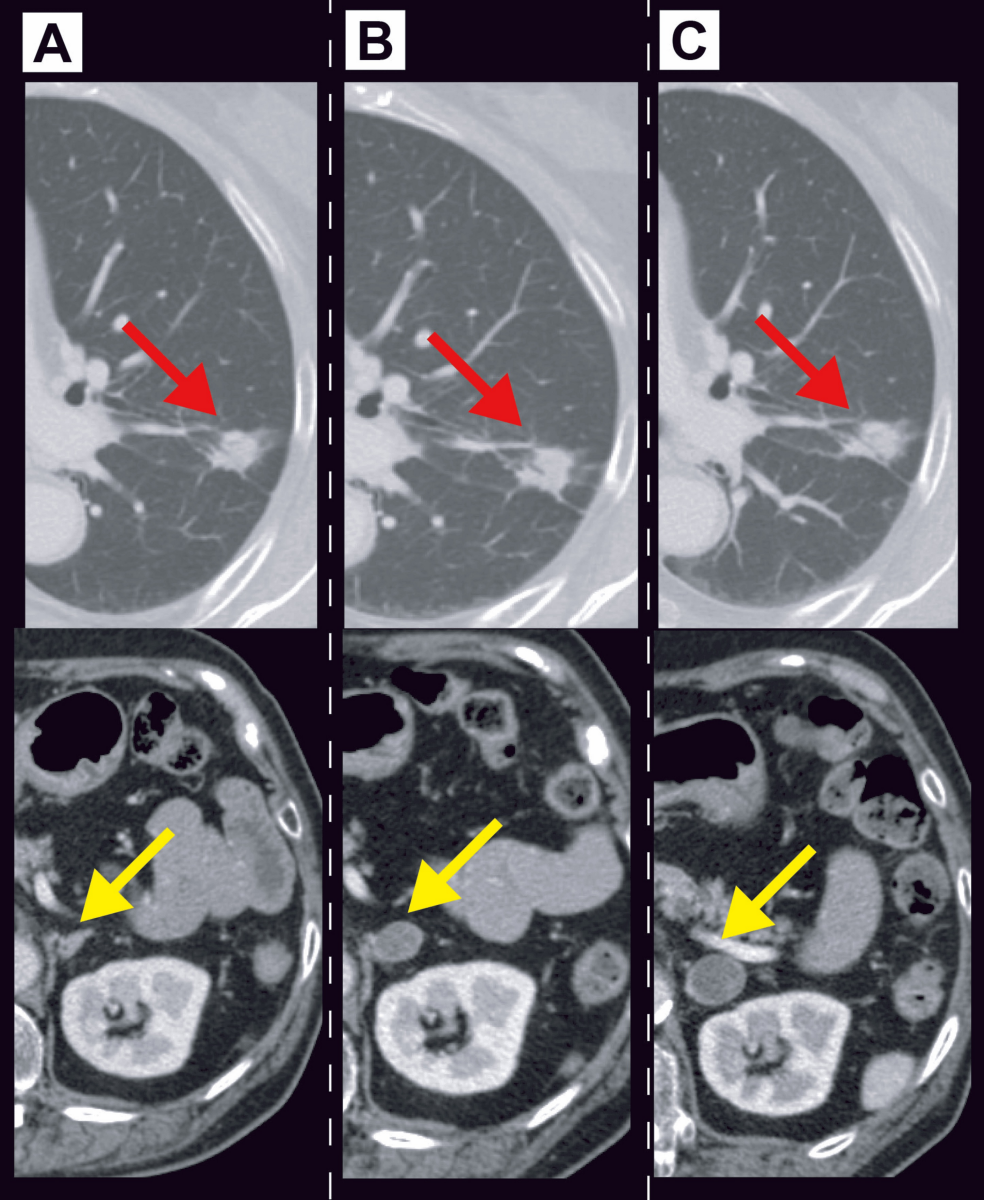
Serial changes in imaging findings of the primary lung tumor and adrenal metastasis A: At the completion of second-line therapy (six months before admission). B: After three cycles of atezolizumab maintenance therapy (three months before admission). C: After six cycles of atezolizumab maintenance therapy (two weeks before admission). The upper panels show serial CT images of the primary lung lesion (red arrows), and the lower panels show those of the adrenal metastasis (yellow arrows). After the initiation of maintenance therapy, the size of the primary tumor remained unchanged, whereas the left adrenal metastasis appeared after three cycles of maintenance therapy and subsequently increased in size.

Evaluation CT after the third cycle of maintenance therapy revealed a heterogeneous 17-mm nodule in the left adrenal gland, which increased to 20 mm by the sixth cycle (Figure 2). Based on these findings, local recurrence due to adrenal metastasis was diagnosed, and the patient was admitted for treatment. At admission, her performance status was equivalent to 1, with stable blood pressure and respiratory status. The abdomen was flat and soft, with no evident tenderness. Laboratory findings showed no significant elevation of inflammatory markers, preserved renal function, and tumor markers within the normal range (Table 1).

**Table 1:** Laboratory findings on admission WBC: white blood cell; Neu: neutrophil; Lym: lymphocyte; Mono: monocyte; Eos: eosinophil; Baso: basophil; RBC: red blood cell; Hb: hemoglobin; Hct: hematocrit; Plt: platelet; Alb: albumin; AST: aspartate aminotransferase; ALT: alanine aminotransferase; LDH: lactate dehydrogenase; ALP: alkaline phosphatase; γ-GTP: gamma‑glutamyl transpeptidase; T-Bil: total bilirubin; Na: sodium; Cl: chloride; K: potassium; BUN: blood urea nitrogen; Cre: creatinine; eGFR: estimated glomerular filtration rate; CRP: C-reactive protein; Ca: calcium; fT3: free triiodothyronine; fT4: free thyroxine; TSH: thyroid stimulating hormone; ACTH: Adrenocorticotropic hormone; CLEIA: chemiluminescent enzyme immunoassay; DHEA-S: dehydroepiandrosterone sulfate; CEA: carcinoembryonic antigen Comprehensive laboratory data obtained at the time of hospital admission, demonstrating slight elevation of renin and plasma aldosterone. Reference ranges are shown in the rightmost column for comparison.

Parameter (Unit)	Value	Reference Range
Blood test		
WBC (/μL)	4900	4500-9000
Neu (%)	64	49-62
Lym (%)	25.8	25-45
Eos (%)	9.2	1-5
Baso (%)	1	0-1
RBC (×10⁴/µL)	371	427-570
Hb (g/dL)	11.6	14-18
Hct (%)	34.8	40-52
Plt (×10⁴/µL)	21.4	15-35
Alb (g/dL)	4.3	3.8-5.3
AST (U/L)	22	8-38
ALT (U/L)	14	4-44
LDH (U/L)	207	106-211
ALP (U/L)	104	104-338
γ-GTP (IU/L)	17	12-63
T-Bil (mg/dL)	0.9	0.3-1.2
Na (mEq/L)	141	135-147
Cl (mEq/L)	105	98-108
K (mEq/L)	4	3.6-5
BUN (mg/dL)	19	8-20
Cre (mg/dL)	0.61	0.61-1.04
eGFR (mL/min)	71.9	60-100
CRP (mg/dL)	0.1	0-0.2
Ca (mg/dL)	9.3	8.6-10.1
fT3 (pg/mL)	2.8	2.3-4
fT4 (ng/dL)	1.05	0.9-1.7
TSH (mIU/mL)	1.67	0.5-5
Cortisol (mg/dL)	15.1	6.4-21
ACTH (pg/mL)	26.4	7.2-63.3
Adrenaline (pg/mL)	23	0-170
Noradrenaline (pg/mL)	279	150-570
Dopamine (pg/mL)	<=20	0-30
Renin activity (ng/mL/h)	5.8	0.2-3.9
Plasma aldosterone:CLEIA (pg/mL)	87.8	4-82.1
DHEA-S (mg/dL)	66	7-177
CEA (ng/mL)	4.8	0-5

Considering that the recurrent lesion was a solitary unilateral adrenal metastasis, and that disease control at all other sites was maintained under ICI maintenance therapy, local therapy was deemed appropriate. Both radiotherapy and surgical resection were considered. However, because the lesion was adjacent to the small intestine, adequate radiation dosing would have been difficult even with stereotactic irradiation. Therefore, surgical resection was judged to provide superior local control.

**Figure 3:**
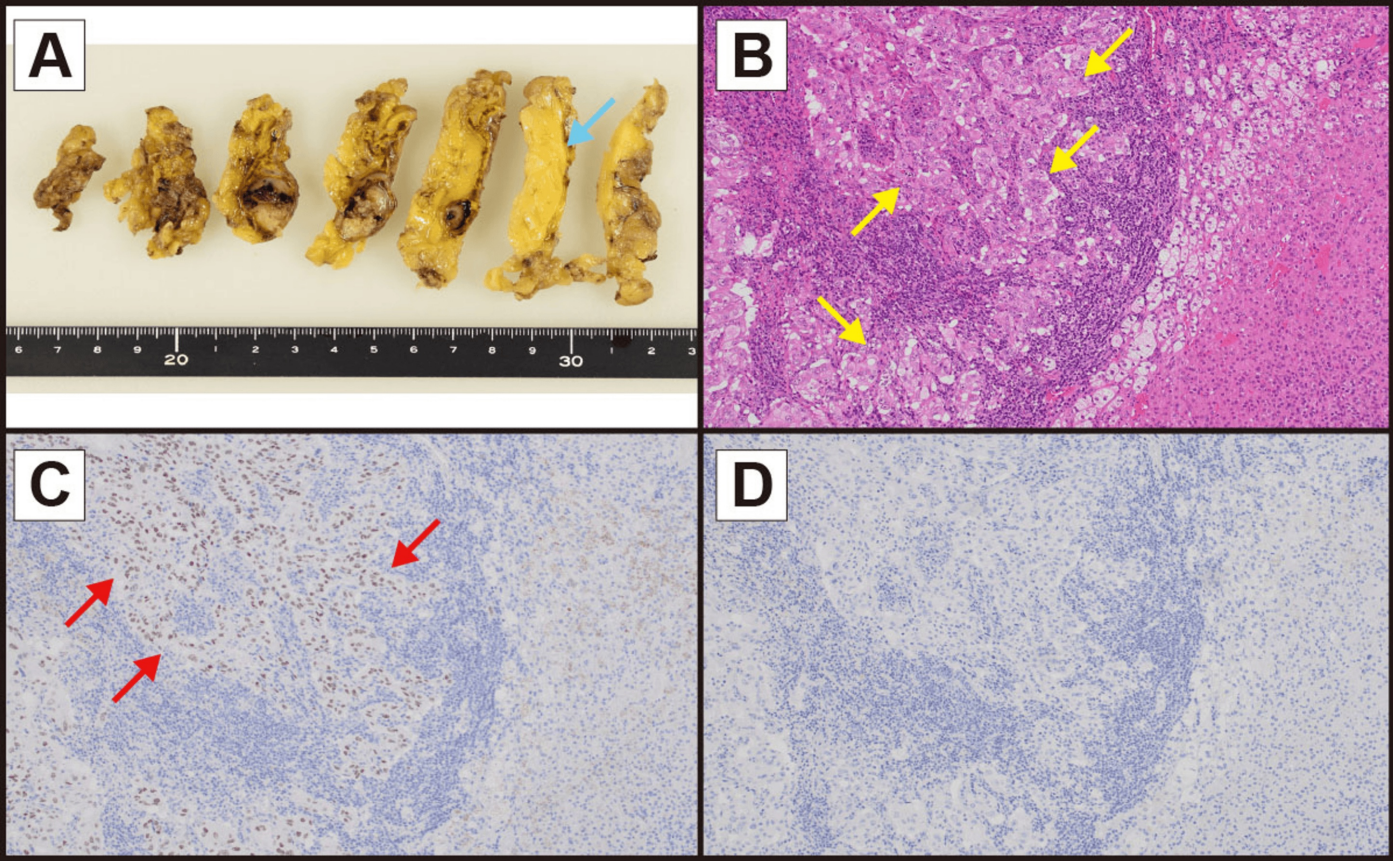
Histopathological images of the resected left adrenal specimen A: A cut surface of the resected left adrenal specimen revealed a white tumor within the adrenal gland (blue arrow). B: Hematoxylin and eosin staining showed tumor cells (yellow arrows) proliferating in a solid papillary pattern. C: Immunohistochemical staining for thyroid transcription factor-1 (TTF-1) demonstrated TTF-1–positive tumor cells (red arrows). D: Immunohistochemical staining for p40 showed no p40-positive tumor cells.

Laparoscopic left adrenalectomy was performed. Histopathological examination of the resected adrenal specimen revealed solid papillary growth of tumor cells positive for thyroid transcription factor-1 (TTF-1) and cytokeratin 7 (CK7), and negative for cytokeratin 20 (CK20) and p40 (Figure 3). Given that the primary lung tumor was TTF-1-positive and p40-negative adenocarcinoma, the findings were consistent with adrenal metastasis from lung cancer. The postoperative course was uneventful, and maintenance atezolizumab was resumed 42 days after surgery. The patient remained recurrence-free with disease control for four years thereafter.

After 68 cycles of maintenance therapy, enlargement of the primary tumor was observed over the course of one year, and the disease was classified as progressive disease; however, no recurrence was detected in the region surrounding the adrenal gland. The patient was subsequently transitioned to third-line therapy with carboplatin plus pemetrexed and has achieved long-term survival while continuing pemetrexed maintenance therapy.

## Discussion

The present case is notable for achieving more than four years of long-term survival after surgical resection of a solitary left adrenal metastasis that developed during maintenance ICI therapy, followed by resumption of ICI treatment. Gomez et al. reported that the addition of local therapy with surgery or radiotherapy significantly prolonged progression-free survival (PFS) and overall survival (OS) in patients with oligometastatic NSCLC [4,5]. Although the benefit of adding local therapy to systemic treatment has gained attention, the optimal treatment strategy has yet to be established.

In this case, the patient was positive for the EGFR L858R mutation, but the response to first-line osimertinib was limited. It has been reported that progression-free survival with EGFR-tyrosine kinase inhibitor (TKI) therapy is reduced in patients with EGFR mutations and high PD-L1 expression [7]. This phenomenon has been attributed to PD-L1-mediated induction of autophagy via the mitogen-activated protein kinase (MAPK) pathway, leading to primary resistance to EGFR-TKIs [8]. Conversely, adrenal metastases from NSCLC, similar to brain and liver metastases, are considered organs with lower responsiveness to ICIs compared with pulmonary or mediastinal lymph node lesions [3]. Accordingly, isolated progression of adrenal metastasis during ICI therapy, as observed in this case, is not uncommon in clinical practice. Previous reports have suggested that, in cases of lung cancer with a resectable primary tumor, resection of a solitary adrenal metastasis may be associated with favorable survival outcomes [9]. Therefore, if disease control can be achieved at sites other than the adrenal metastasis, local treatment may be considered as a therapeutic option to enable continuation of ICI therapy with the aim of improving overall survival.

Reported factors associated with favorable outcomes following local therapy include: (1) a limited number of metastatic lesions, (2) good control of other disease sites with systemic therapy, (3) good performance status, and (4) a relatively long interval from initial treatment to recurrence [4,5,10,11]. Although EGFR-mutated NSCLC is generally considered to have limited responsiveness to ICI monotherapy or combination therapy [12-14], in the present case, disease control of the primary tumor, lymph node metastases, and pleural dissemination - excluding the adrenal metastasis - was maintained for more than six months. Considering the pace of progression observed during prior osimertinib therapy, it was judged - albeit atypically - that atezolizumab was effectively controlling disease at sites other than the adrenal lesion. Furthermore, given the patient’s favorable general condition, she was considered an appropriate candidate for local therapy.

Both surgical resection and stereotactic body radiotherapy (SBRT) are treatment options for adrenal metastases. SBRT has been reported to achieve high local control rates and is considered advantageous due to its minimally invasive nature [15]. However, because the adrenal glands are anatomically adjacent to the gastrointestinal tract and major vessels, dose limitations may preclude sufficient local control in some cases. In the present case, dose constraints due to proximity to the gastrointestinal tract made adequate local control with radiotherapy difficult, and surgical resection was prioritized. Surgical resection offers the advantages of definitive pathological diagnosis and complete removal of metastatic lesions, and prolonged survival has been reported in patients with solitary adrenal metastasis, good performance status, and a relatively long disease-free interval from initial treatment [11,16,17]. In this patient, the solitary nature of the adrenal metastasis, good systemic disease control under ICI maintenance therapy, favorable performance status, and the expectation of early postoperative resumption of ICI therapy were key factors supporting the decision to perform surgery.

Although local therapy during ICI treatment is expected to potentially offer synergistic effects, evidence remains limited, with benefits suggested mainly in the setting of additional treatment for residual lesions after first-line therapy [10,18]. Furthermore, to the best of our knowledge, only one case of long-term survival following ICI therapy after surgical resection for locally recurrent adrenal metastasis has been reported; however, in that case, the recurrence occurred after surgical resection of the primary tumor [6]. In the present case, long-term disease control was achieved by resuming atezolizumab after local resection, suggesting that local therapy may have complemented or enhanced the therapeutic efficacy of ICIs. These findings indicate that even in stage IV NSCLC with oligometastasis or oligoprogression under ICI therapy, proactive consideration of local treatment may contribute to long-term survival when other disease sites are well controlled.

## Conclusions

We report a case of solitary adrenal oligoprogression that developed during maintenance ICI therapy, in which long-term survival was achieved by re-administration of ICI following surgical resection. Even in cases of localized recurrence during ICI treatment, when durable disease control of other lesions can be expected, the appropriate integration of local therapy may help maximize therapeutic benefit.
